# Potential barriers and facilitators for implementation of an integrated care pathway for hearing-impaired persons: an exploratory survey among patients and professionals

**DOI:** 10.1186/1472-6963-7-57

**Published:** 2007-04-19

**Authors:** Janneke PC Grutters, Frans van der Horst, Manuela A Joore, Hans Verschuure, Wouter A Dreschler, Lucien JC Anteunis

**Affiliations:** 1 Department of Clinical Epidemiology and Medical Technology Assessment, University Hospital Maastricht, PO Box 5800, 6202 AZ Maastricht, The Netherlands; 2 Department of Otorhinolaryngology, Head and Neck Surgery, University Hospital Maastricht, Maastricht, The Netherlands; 3 Department of General Practice, Maastricht University, Maastricht, The Netherlands; 4 Audiological Center, Erasmus Medical Center Rotterdam, Rotterdam, The Netherlands; 5 Department of Clinical and Experimental Audiology, Academic Medical Center, Amsterdam, The Netherlands

## Abstract

**Background:**

Because of the increasing costs and anticipated shortage of Ear Nose and Throat (ENT) specialists in the care for hearing-impaired persons, an integrated care pathway that includes direct hearing aid provision was developed. While this direct pathway is still under investigation, in a survey we examined expectations and potential barriers and facilitators towards this direct pathway, of patients and professionals involved in the pathway.

**Methods:**

Two study populations were assessed: members of the health professions involved in the care pathway for hearing-impaired persons (general practitioners (GPs), hearing aid dispensers, ENT-specialists and clinical audiologists) and persons with hearing complaints. We developed a comprehensive semi-structured questionnaire for the professionals, regarding expectations, barriers, facilitators and conditions for implementation. We developed two questionnaires for persons with hearing complaints, both regarding evaluations and preferences, and administered them after they had experienced two key elements of the direct pathway: the triage and the hearing aid fitting.

**Results:**

On average GPs and hearing aid dispensers had positive expectations towards the direct pathway, while ENT-specialists and clinical audiologists had negative expectations. Professionals stated both barriers and facilitators towards the direct pathway. Most professionals either supported implementation of the direct pathway, provided that a number of conditions were satisfied, or did not support implementation, unless roughly the same conditions were satisfied. Professionals generally agreed on which conditions need to be satisfied. Persons with hearing complaints evaluated the present referral pathway and the new direct pathway equally. Many, especially older, participants stated however that they would still visit the GP and ENT-specialist, even when this would not be necessary for reimbursement of the hearing aid, and found it important that the ENT-specialist or Audiological Centre evaluated their hearing aid.

**Conclusion:**

This study identified professional concerns about the direct pathway for hearing-impaired persons. Gaps exist in expectations amongst professions. Also gaps exist between users of the pathway, especially between age groups and regions. Professionals are united in the conditions that need to be fulfilled for a successful implementation of the direct pathway. Implementation on a regional level is recommended to best satisfy these conditions.

## Background

An important step in an implementation process is to determine the barriers that will impede implementation, and the facilitators that will enhance implementation, within different subgroups [1-5]. Insight into barriers and facilitators will help to design the most effective implementation strategy. In the Netherlands a new care pathway for hearing-impaired adults is currently under investigation. The present study focuses on potential barriers and facilitators for implementation of this new care pathway.

**Figure 1:**
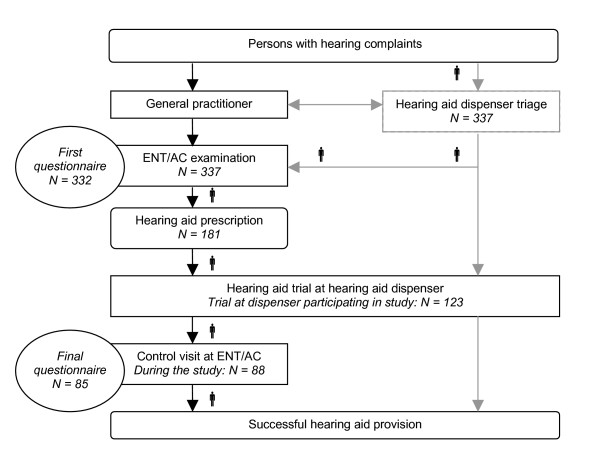
**Graphical representation of referral pathway^1 ^and the direct pathway^2^, including the study pathway^3^, the study population^4 ^and the questionnaire timing^5^**. ^1 ^Referral pathway is illustrated with black lines. ^2 ^Direct pathway is illustrated with black and grey lines. ^3 ^Study pathway is illustrated with . ^4 ^Study population is represented in italic. ^5 ^Questionnaire timing is illustrated with rounds.

Most hearing-impaired adults have an age-related sensorineural hearing loss, where hearing aids are the only option for rehabilitation. Until recently, in the Netherlands all hearing-impaired persons who seek help first had to consult their general practitioner (GP) for referral to an Ear Nose and Throat (ENT) specialist or Audiological Centre (AC). In an AC a multidisciplinary team under the direction of a clinical audiologist offers specialized care for the more serious or complicated hearing problems. Only ENT-specialists and ACs were entitled to prescribe hearing aids. Generally, a hearing aid dispenser performed the actual hearing aid fitting. Once a satisfying hearing aid was fitted, the hearing-impaired persons returned to the ENT-specialist or AC for approval of the hearing aid, which entitled them to (partial) reimbursement by their medical insurance. We will refer to this pathway as the care pathway for hearing-impaired persons by referral (Figure 1), in short the *referral *pathway.

Since 2002 however a number of health insurance companies in the Netherlands permit hearing aid reimbursement without a referral, prescription or approval. This allows for direct hearing aid provision by the hearing aid dispenser. One reason for this new care pathway was to reduce health care costs. Approximately 10% of the general population of western countries is hearing impaired, and this prevalence heavily increases with age [6,7]. As a result the current growth of the ageing population causes a proportional growth of the population with hearing impairment. This will raise medical consumption for hearing impairment and, as a consequence, increases the costs of medical care. Another reason for the new care pathway was to reduce the workload of ENT-specialists, since a report was published in 2000 that alerted to a future shortage of ENT-specialists in the Netherlands [8].

It has been suggested that a proportion of hearing-impaired individuals can safely be fitted with a hearing aid without medical care [9-12]. These individuals are defined as clients throughout the present study. In order to distinguish these 'clients' from the 'patients' who require medical attention or treatment of an AC, a set of criteria was developed by a national body of professionals involved in the care pathway for hearing-impaired persons [13]. The criteria for referral relate to otoscopy (i.e. skin aberration, fluid in the auditory canal), audiometry (i.e. asymmetrical hearing loss or sudden deafness) and psychosocial aspects (i.e. communication problems at work). This set of criteria is part of a recently developed integrated care pathway for hearing-impaired persons. Integrated care pathways are structured multidisciplinary care plans which detail essential steps in the care of patients with a specific problem [14]. The new integrated care pathway is referred to as the direct care pathway for hearing-impaired persons including direct hearing aid provision, in short the *direct *pathway (Figure 1). In parts of the United Kingdom, direct hearing aid provision is by now daily routine [15]. While in the Netherlands the safety and efficiency of the direct pathway are still under evaluation, we found it important to examine whether persons with hearing complaints and professionals support the direct pathway, and what they consider to be barriers and facilitators, in order to anticipate on the implementation process of the direct care pathway.

The objective of this study was therefore to gain insight into the expectations and potential barriers and facilitators towards the direct pathway.

## Methods

### Study population

Two study populations were assessed in the present study. The first study population consisted of members of the health care professions involved in the care pathway for hearing-impaired persons: GPs, hearing aid dispensers, ENT-specialists and clinical audiologists. We approached professionals from three regions in the Netherlands (Amsterdam, Maastricht and Rotterdam), divided over the four professions.

The second study population consisted of persons who consulted for hearing complaints. We included persons of 50 years and older with hearing complaints, who consulted ENT departments and ACs or hearing aid dispensers in the three regions. Persons who had consulted for hearing complaints in the preceding year were considered to be under treatment and were therefore excluded.

### Design

Data for this exploratory survey were collected as part of a multi-centre non-controlled prospective evaluation study examining the direct pathway versus the referral pathway [16]. The study was carried out from April 2004 until August 2005 in three regions (Amsterdam, Maastricht and Rotterdam) in the Netherlands. The study was approved by the Medical Ethical Committees of the participating hospitals, and written consent was obtained from all participants. Three teaching hospitals and 11 hearing aid dispensers participated in the study. Since the hospital in Maastricht is the only hospital in its region, it also has a community function. The hospitals in Amsterdam and Rotterdam mainly operate as centres for secondary referral for patients in need of specialized care.

The study focused on two key elements of the direct care pathway: distinguishing persons who need medical or specialized audiological care (the patients) from persons who do not need medical or specialized audiological care (the clients), and the quality of the hearing aid provision. For the purpose of the study, participants first visited the hearing aid dispenser for a triage visit (dispenser-triage, Figure 1). At the triage visit, the hearing aid dispenser examined whether the participant was in need of medical or specialized audiological care or not and hence should be defined as a patient or client respectively. To perform this examination, the hearing aid dispenser had followed a training program especially designed for this purpose. The main topics of the training program were audiometry and otoscopy. Regardless of the outcome of the triage, all participants subsequently visited the ENT-specialist and AC for a second examination (ENT/AC-examination), to evaluate whether the dispenser had correctly defined the participant as patient or client. If the participant received a prescription for hearing aids, the hearing aid dispenser fitted a hearing aid. Once a satisfying hearing aid fitting was obtained, the participant returned to the AC for a control visit, to evaluate the quality of the hearing aid fitting.

### Framework

Barriers and facilitators are defined as factors that prevent or enhance, respectively, behavioural change [5]. Various theories and models for change point to a multitude of factors that may affect change [4,17]. Cabana et al [18] developed a framework that focuses on the individual professional. In this framework barriers are classified into three main categories: barriers related to knowledge (familiarity, awareness), barriers that affect attitudes (agreement, self-efficacy, motivation, outcome expectancy) and external barriers (patient factors, guideline factors, environmental factors). We used this framework to structure the barriers and facilitators that health care professionals perceived regarding the implementation of the direct pathway.

In the care pathway for hearing-impaired persons, patient factors play an important role as well. Therefore, patient factors were examined separately in a survey among persons with hearing complaints. Wensing and Elwyn [19] distinguish three types of patient views: evaluations (reactions to their service experience), preferences (ideas about what should occur) and reports (objective observations). In this study we focused on subjective patient views, and therefore examined evaluations and preferences.

### Questionnaire for professionals

Health care professionals involved in the care pathway (GPs, hearing aid dispensers, ENT-specialists and clinical audiologists) were asked to complete a comprehensive semi-structured questionnaire regarding the direct pathway [see Additional file 1]. First, we paid explicit attention to the outcome expectancies. Professionals were asked on five point Likert scales whether they expected the direct pathway to have positive or negative consequences for different aspects of the hearing aid provision. These six aspects were: diagnosing hearing impairment; indicating a hearing aid; paying attention to medical, audiological and psychological complications; giving objective information about the impairment, treatment options and hearing aids; choice and fine-tuning of the hearing aid; and the quality of the ear moulds. Next, using open questions the professionals were asked to report if they thought the direct pathway was an improvement for some hearing-impaired persons, and for whom, and whether it was a deterioration for some hearing-impaired persons, and for whom. They were also asked to report possible risks and benefits of the direct pathway. Finally, professionals were asked if the direct pathway should be implemented and, if applicable, under which conditions.

### Questionnaire for persons with hearing complaints

Two questionnaires for persons with hearing complaints, designed for self-completion, were developed. The questionnaires were administered after the participants had experienced the two key elements of the direct pathway: the triage and the hearing aid fitting. The first questionnaire was administered directly after the ENT/AC-examination [see Additional file 2]. Two questions concerned evaluations of the hearing aid dispenser: whether participants had confidence that the hearing aid dispenser, who had received additional training, was able to distinguish between clients and patients, and whether they were satisfied with the way the hearing aid dispenser behaved towards them. Regarding their preferences, we asked the participants if they would be inclined to obtain a hearing aid sooner when they didn't have to visit their GP and an ENT-specialist or AC first. Also, they were asked whether they would still visit the GP and ENT-specialist or AC if this was not a prerequisite for (partial) reimbursement of the hearing aid. The questions were answered on five point Likert scales and participants were also given the option that they did not know.

As our study population consisted of persons with hearing complaints, and not only hearing aid applicants, not all participants had a hearing aid fitted. The subset of persons with hearing complaints who were fitted with a hearing aid within the study period completed the final questionnaire after the control visit at the AC [see Additional file 3]. Both the referral pathway and the direct pathway were described and participants were asked to value both pathways with a grade between 1 and 10, with 10 being the most favourable. After these two evaluative questions, three preference questions were asked on five point Likert scales, including a 'don't know' option. Participants were asked whether they expected that the hearing aid dispenser would fit a good hearing aid without involvement of the ENT-specialist or AC and to report if they found it important that the hearing aid fitting would be advised and evaluated by the ENT-specialist or AC.

We checked the results for differences in sex (male versus female) and age group (below versus above median age) using Mann-Whitney U tests. Differences between the regions (Amsterdam, Maastricht, Rotterdam) were examined with Kruskal Wallis tests, and Mann-Whitney U tests for post-hoc pairwise comparisons.

## Results

### Professional views

#### Outcome expectancy

A total of 60 health care professionals completed the questionnaire regarding their expectations towards the direct pathway. This group consisted of 14 GPs, 16 hearing aid dispensers, 16 ENT-specialists and 14 clinical audiologists.

**Figure 2:**
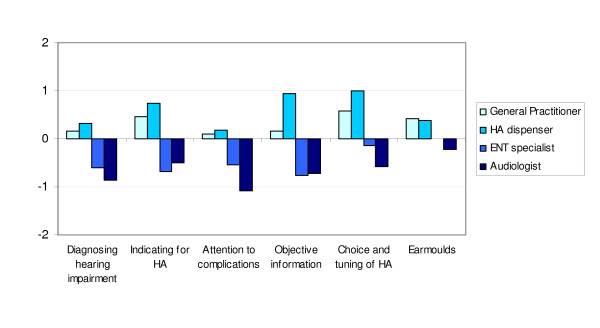
Expectations of profession groups towards the direct pathway*. * Elicited with five point Likert scales, ranging from 2 (positive) to -2 (negative)

The professional groups gave significantly different answers on all aspects: diagnosing hearing impairment; indicating a hearing aid; paying attention to medical, audiological and psychological complications; giving objective information about the impairment, treatment options and hearing aids; choice and fine-tuning of the hearing aid; and the quality of the ear moulds (Kruskal Wallis, p-value < 0.01). On average GPs and hearing aid dispensers expected the direct pathway to have positive consequences for all aspects (Figure 2). The ENT-specialists expected on average negative consequences for all aspects except the quality of the ear moulds, for which they expected no change. The clinical audiologists expected on average negative consequences for all six aspects.

#### Barriers and facilitators

All professionals considered the direct pathway an improvement for a subpopulation of hearing-impaired persons (Table 1). Professionals defined this subpopulation as 'older' persons, persons with hearing loss due to ageing only, persons with initial resistance for seeking medical care, and experienced hearing aid users. Almost all professionals (98%) saw benefits in the direct pathway. These benefits were breaking down barriers, efficiency gain, quicker access to medical care for those in need of it, minimization of costs, reduction in the duration of the pathway, and reduction of the burden on the ENT-specialist, resulting in more time for medical specialist care. Hearing aid dispensers also reported closer collaboration between the involved professions as a benefit.

**Table 1:** Opinions of professionals on improvement, deterioration, risks and benefits of the direct pathway

Profession group	Valid N	Improvement? % Yes	Benefits? % Yes	Deterioration? % Yes	Risks? % Yes
GP	14	100%	100%	64%	79%
HA dispenser	16	100%	100%	56%	63%
ENT- specialist	16	100%	94%	94%	100%
Clinical audiologist	14	100%	100%	100%	93%
Total	60	100%	98%	78%	83%

The majority of professionals (78%) on the other side considered the direct pathway a deterioration for persons in need of medical care, 'younger' persons, and persons who are dissatisfied with their hearing aid. The majority of professionals (83%) mentioned the risk of undetected pathology, wrongful indication for a hearing aid or commercialization of care as potential risks of the direct pathway. In addition, GPs emphasized that their role as gatekeeper should be maintained. They reported that a medical cause of the hearing problems should be ruled out first and that diagnostics and treatment should not go hand in hand. By this they meant that the hearing aid dispenser should not both diagnose hearing impairment and provide the hearing aid. Clinical audiologists additionally reported that both the GP and the hearing aid dispenser are not capable of distinguishing adequately between patients and clients.

#### Conditions for implementation

**Figure 3:**
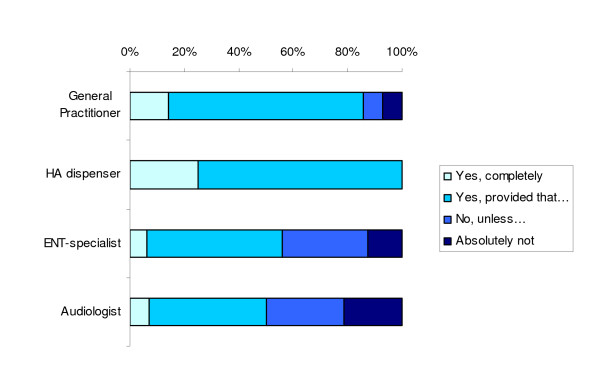
Responses to the question whether professionals support implementation of the direct pathway.

Ten out of 14 GPs stated that they supported the direct pathway, provided that specific conditions were met (Figure 3). These conditions mostly concerned their own role in the pathway: GPs wanted to stay involved in the direct care pathway and emphasized the importance of a good communication between hearing aid dispensers and themselves. Other conditions were adequate training for hearing aid dispensers, that direct hearing aid provision should only be possible for persons of 65 years and older, and that, especially in the beginning, the care should be evaluated frequently.

Two GPs completely supported implementation. One GP reported not to support implementation, unless GP and hearing aid dispenser would work together, and one GP reported absolutely not to support implementation.

Twelve out of 16 hearing aid dispensers reported that they supported implementation, but only when adequate training, strict requirements for hearing aid dispensers, clear criteria for referral, excellent communication between professionals, and good arrangements regarding task division were provided. Four hearing aid dispensers reported to support the implementation completely.

Eight out of 16 ENT-specialists stated to support implementation of the direct pathway, provided that the quality of audiometry and otoscopy would be sufficient, that hearing aid dispensers would not prescribe hearing aids for commercial reasons only, that the professional education for hearing aid dispenser would improve, and that clear criteria for referral, a good evaluation system and a complaints service would be present. Five ENT-specialists reported not to support implementation, unless some of the above mentioned conditions were satisfied. Two ENT-specialists did not support implementation and one ENT-specialist reported to completely support implementation of the direct pathway.

Six out of 14 clinical audiologists stated that they did support implementation, provided that quality would be guaranteed, that there would be a second-opinion possibility, that task division, responsibilities and criteria for referral would be clear, and that hearing aid dispensers would be adequately trained for their new tasks. Four clinical audiologists did not support implementation, except when hearing aid dispenser and GP would recognize all pathology, hearing aid dispensers were sufficiently trained, and a random evaluation of the care pathway would exist. Three clinical audiologists did absolutely not support implementation, in contrast to one who completely supported implementation.

### Views of persons with hearing complaints

#### Study population

Between April 2004 and August 2005, a total of 337 persons with hearing complaints were included in the study and were asked to complete the first questionnaire after the ENT/AC-examination. A total of 332 participants (99%) completed the questionnaire after the ENT/AC-examination (Figure 1). Their mean age was 69.6 years (sd 9.0; median 70) and 60% were male. The participants were included in Amsterdam (n = 75), Maastricht (n = 186) and Rotterdam (n = 71).

Of the 337 participants in the study, 181 (54%) had a hearing aid fitted. Of them, 88 (49%) attended the AC for the control visit after the hearing aid trial. Ninety-three participants (51%) who had a hearing aid fitted did not show up at the control visit. These participants had not finished their hearing aid trial before the end of the study (n = 35), or had a hearing aid fitted at a different dispenser that not participated in the study (n = 58) and therefore dropped out of the study. Of the 88 participants who did attend to the control visit, 85 (97%) completed the final questionnaire. These 85 participants had a mean age of 70.6 years (sd 7.8; median 71) and 72% were male. The participants were included in Amsterdam (n = 14), Maastricht (n = 53) and Rotterdam (n = 18).

#### Evaluations

After the ENT/AC-examination, 89% of the persons with hearing complaints reported to have confidence in the trained hearing aid dispenser (Table 2). Even more respondents (96%) were satisfied with the way the hearing aid dispenser behaved towards them.

**Table 2:** Evaluations and preferences of persons with hearing complaints

Question	Valid N	Yes	Neutral	No	Don't know
*Evaluations after ENT/AC examination (N = 332)*					
Confidence in hearing aid dispenser?	330	89%	5%	2%	4%
Satisfied with hearing aid dispenser?	331	96%	2%	2%	0%
					
*Preferences after ENT/AC examination (N = 332)*					
Earlier fitted with a hearing aid without GP/ENT/AC visit?	328	24%	21%	52%	3%
Visit GP with hearing complaints if not necessary?	329	54%	12%	32%	2%
Visit ENT/AC with hearing complaints if not necessary?	329	64%	13%	21%	3%
					
*Preferences after hearing aid fitting (N = 85)*					
Expect dispenser to fit good hearing aid without mediation ENT/AC?	85	80%	12%	6%	2%
Important to be advised by ENT/AC?	84	60%	21%	17%	2%
Important that hearing aid is evaluated by ENT/AC?	83	64%	17%	19%	0%

The subset of persons with hearing complaints who had finished their hearing aid trial and therefore completed the whole pathway (n = 85), completed the final questionnaire after the AC control visit. They graded the referral pathway and the direct pathway equally (7.6 and 7.7 respectively). Both scores ranged from 2 to 10, with a median of 8 for both pathways. The grades were not significantly different (Wilcoxon Signed Ranks Test, p-value 0.896).

#### Preferences

In the first questionnaire, a quarter of the persons with hearing complaints (24%) reported that they would be inclined to obtain a hearing aid sooner when they didn't have to visit their GP, an ENT-specialist or AC first (Table 2). This indicates that a quarter of the participants consider the visits to GP, ENT-specialist and AC as a barrier for hearing aid fitting. Of the participants, 32% would not visit the GP and 21% would not visit the ENT-specialist and AC with their hearing complaints when this was not necessary for reimbursement of the hearing aid.

In the final questionnaire, 80% of the subset of participants who had a hearing aid fitted expected the hearing aid dispenser to perform a good hearing aid fitting, even when the ENT-specialist and AC would not be involved in the process of hearing aid fitting. Even though, a majority of the participants reported that they found it important that an ENT-specialist or AC would advise them (60%), and found it important that the ENT-specialist or AC evaluated the hearing aid fitting (64%).

#### Differences between groups

Additionally, we checked the results for differences in sex (male versus female), age group and region (Amsterdam, Maastricht, Rotterdam). The age groups were defined below and above the median age, being 70 for the group with hearing complaints that completed the first questionnaire, and 71 for the group with a hearing aid fitting that completed the second questionnaire. This resulted in a comparison of persons younger than 70 versus 70 years and older for the first questionnaire, and a comparison of persons younger than 71 versus 71 years and older for the second questionnaire.

No statistically significant differences were found between men and women. There were differences in preferences between the age groups. Of the group of persons aged 70 years and older, 69% would still visit the ENT-specialist or AC, even if this was not necessary for reimbursement of the hearing aid, versus 58% of those younger than 70 years. This difference is statistically significant (Mann-Whitney U, p-value 0.041). Also, statistically significantly more persons of 71 years and older (79%) than persons younger than 71 (49%) found it important that an ENT-specialist or AC evaluated their hearing aid fitting (Mann-Whitney U, p-value 0.005).

We also found differences in preferences for the regions, specifically in whether respondents would still visit their GP when this was not necessary (Kruskal Wallis Test, p-value 0.005). Pairwise comparison showed that in Maastricht (60%) and Amsterdam (57%) statistically significantly more persons stated that they would still visit the GP for their hearing complaints than in Rotterdam (37%) (Mann Whitney U, p-values 0.001 and 0.019).

## Discussion

This exploratory study provides insight in the barriers and facilitators of implementing the direct care pathway for hearing-impaired persons. Hearing aid dispensers and GPs had on average positive expectations towards the direct pathway, while ENT-specialists and clinical audiologists had on average negative expectations. Also more ENT-specialists and clinical audiologists found the direct pathway a deterioration and stated possible risks, and they were somewhat more reluctant to implement the direct pathway. Besides concerns about safety and quality, a possible explanation for this is that in the direct pathway, ENT-specialists and clinical audiologists will lose domain in the health care for hearing-impaired persons. They have to hand over part of their work to the GP and hearing aid dispenser. They may feel that their professional authority will decline if they are relegated more to the role of specialist, seeing patients only on referral from hearing aid dispensers and GPs. Hearing aid dispensers on the other hand experience an expansion of their tasks in the care for hearing-impaired persons, as they will have greater autonomy in the direct pathway. This makes hearing aid dispensers a so-called 'encroaching profession'. Also in other health care settings established professions develop strategies to protect their boundaries, while encroaching professions try to expand their work areas [20-23].

In 2005 an updated report regarding the labour market of ENT-specialists was published. This report concluded that the predicted shortage of ENT-specialists was tackled, and that a sufficient number of ENT-specialists will be available in the near future [24]. These findings possibly play a role in the expectations of especially the ENT-specialists, since they may be more reluctant to hand over a number of tasks when their workload will not be as heavy as they expected it to be. The latter also happened in the field of orthopaedic surgeons, who decided to discard a number of unwanted tasks when there was an undersupply of orthopaedic surgeons, and wished to reclaim these tasks when an oversupply arose [21].

Although negative expectations exist, the results of this study show that most health care professionals either supported implementation of the direct pathway, provided that a number of conditions are satisfied, or did not support implementation, unless roughly the same conditions are satisfied. The professionals generally agreed on which conditions need to be satisfied. In general, these conditions are: good communication between the professionals involved in the direct care pathway, adequate training for the hearing aid dispensers, frequent evaluation, clear criteria for referral, clear division of tasks, a second opinion possibility, and a complaints service.

Before implementing the direct care pathway, all parties involved (GPs, hearing aid dispensers, ENT-specialists, clinical audiologists and hearing-impaired persons) should reach a consensus on the criteria for referral and the division of tasks. Regardless of these criteria and the division of tasks, it should always be possible for hearing-impaired persons to visit the ENT-specialist or AC for a second opinion. Furthermore, the parties must agree on clear requirements regarding the training of the hearing aid dispensers, and these requirements must concur with the referral criteria and task description of the hearing aid dispensers. Next, a complaints service should be facilitated, possibly by the patients' association. Especially in the beginning the direct care pathway should be evaluated carefully. Good communication between professionals is of great importance, but this is probably not easy to realize. Regional implementation may be part of the solution, as in small regions it is less difficult to create a basis of trust among the professionals involved. When the professionals and hearing-impaired persons make agreements on a regional level, they will get to know each other better. As a result this might improve their communication and their confidence in the other professionals' capabilities.

All four groups of professionals stated that the direct pathway had risks as well as benefits. When we apply the framework of Cabana et al [18] to the barriers mentioned by the professionals, all are barriers that affect attitudes. The GPs mentioned barriers related to lack of agreement, being the importance of their gatekeeper role and that diagnostics and treatment should not go hand in hand. Most barriers however are related to lack of outcome expectancy. This is clear from the negative expectations of ENT-specialists and clinical audiologists (Figure 2) towards the direct pathway, but also shows in the risks that were stated by all professionals. Professionals stated the risk of undetected pathology, the risk of wrongful indications for a hearing aid, and the risk of fitting hearing aids for commercial reasons only. Some professionals considered the GP and hearing aid dispenser not capable of distinguishing between persons who need medical or specialized audiological care (patients) and persons who do not need medical or specialized audiological care (clients). If the direct pathway will be implemented, it is important to first consider and appropriately influence the attitudes of the professionals. This may be more successful on a regional level, since closer collaboration between the professionals will probably result in more confidence in each other's capabilities. Information with regard to the safety of the direct pathway is likely to be crucial. Although barriers related to knowledge were not mentioned in this study, when implementing the direct pathway it is important to make sure that everyone involved has sufficient knowledge of the pathway.

The professionals did not perceive any external barriers, but we examined these separately by asking persons with hearing complaints for their evaluations and preferences. Due to the study design, approximately half of the participants fitted with a hearing aid could not be asked to complete the final questionnaire. We checked whether age and region in this group were different from the group that did complete the final questionnaire, as these characteristics were found to influence preferences. Since there was no statistically significant difference, we had no reason to believe that the low response rate of the final questionnaire influenced the results.

Although participants evaluated both pathways equally and had confidence in the hearing aid dispenser, many participants stated that they would still visit the GP and ENT-specialist, even when this would not be necessary for reimbursement. This means that after implementation of the direct pathway, clients may still choose to visit the ENT-specialist, bypassing the direct pathway. It is therefore important that persons with hearing complaints are well informed about the direct pathway and are involved in the implementation process.

A larger proportion of older participants stated that they would visit the ENT-specialist or AC, even when this was not necessary for reimbursement of the hearing aid, and found it important that the ENT-specialist or AC evaluated their hearing aid fitting. This indicates that older persons are more attached to the opinion of the medical specialist. This difference was not caused by differences in hearing aid experience. The implication of this difference is that especially older clients may bypass the direct care pathway by still visiting the ENT-specialist or AC for their examination and control visit. Older persons therefore may need special attention in the implementation process. Differences in preferences were found between the regions, which is probably due to the variety in care pathways for hearing-impaired persons that currently exist among regions. Since 2002 a number of health insurance companies already permit some form of direct hearing aid provision without consultation of the involved professionals. This makes the current care for hearing-impaired persons diffuse. The differences between the regions and the current diffuse forms of care for hearing-impaired persons argue for implementation on a regional level. The different types of hospitals may have caused the difference in participant numbers included in the study. Maastricht included more participants than Rotterdam and Amsterdam, possibly because the hospital in Maastricht also has a community function and sees more persons that are not referred by another hospital.

Since multidisciplinary collaboration and task substitution play an important role in many other integrated or shared care pathways [14,23,25,26], the barriers and conditions for implementation found in this study are likely to apply to other integrated care pathways as well.

While the direct care pathway is still under investigation, we have already obtained valuable information regarding support of the direct care pathway and potential barriers and facilitators. Barriers and facilitators to change are often examined after or during implementation [18,27-29]. By determining barriers in an earlier stage, we can use the information to develop the implementation process and to increase the chance of successful implementation.

## Conclusion

This exploratory study identified professional concerns about the direct care pathway for hearing-impaired persons. It is clear that gaps in expectations amongst GPs, hearing aid dispensers, ENT-specialists and clinical audiologists exist. It is also clear that gaps in expectations exist among the users of the care pathway, especially with older persons demonstrating a preference for the traditional pathway. Also, persons from different regions where currently different pathways exist demonstrated different preferences. Despite these differences and despite the fact that implementation of the direct pathway is not yet broadly based, professionals are united in the conditions that need to be fulfilled for a successful implementation of the direct pathway. It is important that these conditions are met before implementing the direct pathway in the Netherlands. Implementation on a regional level is recommended to best satisfy the stated conditions.

## Competing interests

The author(s) declare that they have no competing interests.

## Authors' contributions

All authors have participated in designing the study. JG contributed to the acquisition of the data, drafted the manuscript, performed the statistical analyses and interpreted the data. FVDH, MJ and LA contributed to the acquisition and interpretation of the data and were involved in drafting the manuscript. WD and HV contributed to the acquisition of the data and provided critical edits to the manuscript. All authors have given final approval of the submitted manuscript.

## Pre-publication history

The pre-publication history for this paper can be accessed here:



## Supplementary Material

Additional File 1 Questionnaire for professionals Click here for file

Additional File 2 First questionnaire for persons with hearing complaints Click here for file

Additional File 3 Final questionnaire for persons with hearing complaints Click here for file
