# Supplementary material for: Potential barriers and facilitators for implementation of an integrated care pathway for hearing-impaired persons: an exploratory survey among patients and professionals
Source: BMC Health Serv Res. 2007 Apr 19;7:57. doi: 10.1186/1472-6963-7-57 (PMC1865538; doi:10.1186/1472-6963-7-57)
Supplement: Additional File 1 — Questionnaire for professionals [file 1472-6963-7-57-S1.doc]

| Questionnaire for professionals | | | | | | | | | | | | | | |
| --- | --- | --- | --- | --- | --- | --- | --- | --- | --- | --- | --- | --- | --- | --- |
|  | | | | | | | | | | | | | | |
| What consequences do you think that the new care pathway for hearing-impaired persons will have with regard to the following aspects: | | | | | | | | | | | | | | |
|  | | | | | | | | | | | | | | |
| 1. Diagnosing hearing impairment? | | | | | | | | | | | | | | |
|  | -1q | | -2q | | 0q | | | | 1q | | 2q | |  | |
| Negative consequences | | | | | | | Positive consequences | | | | | | | |
|  | | | | | | | | | | | | | | |
| 1. Indicating a hearing aid? | | | | | | | | | | | | | | |
|  | | -1q | | -2q | | 0q | | | | 1q | | 2q | |  |
| Negative consequences | | | | | | | | Positive consequences | | | | | | |
|  | | | | | | | | | | | | | | |
| 1. Paying attention to medical, audiological and psychological complications? | | | | | | | | | | | | | | |
|  | | -1q | | -2q | | 0q | | | | 1q | | 2q | |  |
| Negative consequences | | | | | | | | Positive consequences | | | | | | |
|  | | | | | | | | | | | | | | |
| 1. Giving objective information on hearing impairment, treatment options and hearing aids? | | | | | | | | | | | | | | |
|  | | -1q | | -2q | | 0q | | | | 1q | | 2q | |  |
| Negative consequences | | | | | | | | Positive consequences | | | | | | |
|  | | | | | | | | | | | | | | |
| 1. Choice and fine-tuning of the hearing aid? | | | | | | | | | | | | | | |
|  | | -1q | | -2q | | 0q | | | | 1q | | 2q | |  |
| Negative consequences | | | | | | | | Positive consequences | | | | | | |
|  | | | | | | | | | | | | | | |
| 1. The quality of the ear moulds? | | | | | | | | | | | | | | |
|  | | -1q | | -2q | | 0q | | | | 1q | | 2q | |  |
| Negative consequences | | | | | | | | Positive consequences | | | | | | |

| 1. Do you think the new care pathway will be an improvement for a subset of persons? | | | | | | |
| --- | --- | --- | --- | --- | --- | --- |
|  | | ❑ | | Yes, that is… |  | |
|  | | ❑ | | No, because… |  | |
|  | | | | | | |
| 1. Do you think the new care pathway will be a deterioration for a subset of persons? | | | | | | |
|  | | ❑ | | Yes, that is… |  | |
|  | | ❑ | | No, because… |  | |
|  | | | | | | |
| 1. Do you think the new care pathway will have possible risks? | | | | | | |
|  | | ❑ | | Yes, that is… |  | |
|  | | ❑ | | No, because… |  | |
|  | | | | | | |
| 1. Do you think the new care pathway will have possible benefits? | | | | | | |
|  | | ❑ | | Yes, that is… |  | |
|  | | ❑ | | No, because… |  | |
|  | | | | | | |
| 1. Do you think that the new care pathway should be implemented? | | | | | | |
|  | ❑ | | Yes, completely | | |  |
|  | ❑ | | Yes, provided that… | | |  |
|  | ❑ | | I don’t know, because… | | |  |
|  | ❑ | | No, unless… | | |  |
|  | ❑ | | Absolutely not | | |  |
