# Supplementary material for: Potential barriers and facilitators for implementation of an integrated care pathway for hearing-impaired persons: an exploratory survey among patients and professionals
Source: BMC Health Serv Res. 2007 Apr 19;7:57. doi: 10.1186/1472-6963-7-57 (PMC1865538; doi:10.1186/1472-6963-7-57)
Supplement: Additional File 2 — First questionnaire for persons with hearing complaints [file 1472-6963-7-57-S2.doc]

# First questionnaire for persons with hearing complaints

| With the following statements we would like to find out how you think about the care for persons with hearing complaints. Could you please indicate whether you agree with the following statements? | | | | | | |
| --- | --- | --- | --- | --- | --- | --- |
|  | Totally agree | Quite  agree | Neither agree nor disagree | Quite disagree | Totally disagree | Don’t know |
| 1. I have confidence that the trained hearing aid dispenser is able to determine whether referral to an ENT-specialist or AC is necessary | ❑ | ❑ | ❑ | ❑ | ❑ | **O** |
|  |  |  |  |  |  |  |
| 1. I am satisfied with the way the hearing aid dispenser behaved towards me | ❑ | ❑ | ❑ | ❑ | ❑ | **O** |
|  |  |  |  |  |  |  |
| 1. I am inclined to obtain a hearing aid sooner, when I don’t have to visit my GP and an ENT-specialist or AC first | ❑ | ❑ | ❑ | ❑ | ❑ | **O** |
|  |  |  |  |  |  |  |
| 1. I would still visit my GP for my hearing complaints if this was not necessary for reimbursement of the hearing aid | ❑ | ❑ | ❑ | ❑ | ❑ | **O** |
|  |  |  |  |  |  |  |
| 1. I would still visit the ENT-specialist or AC for my hearing complaints if this was not necessary for reimbursement of the hearing aid | ❑ | ❑ | ❑ | ❑ | ❑ | **O** |
