# Supplementary material for: Potential barriers and facilitators for implementation of an integrated care pathway for hearing-impaired persons: an exploratory survey among patients and professionals
Source: BMC Health Serv Res. 2007 Apr 19;7:57. doi: 10.1186/1472-6963-7-57 (PMC1865538; doi:10.1186/1472-6963-7-57)
Supplement: Additional File 3 — Final questionnaire for persons with hearing complaints [file 1472-6963-7-57-S3.doc]

| The care for hearing-impaired persons can be organised in different ways. With the next questions we would like to know what you think about two possible ways of organising this care. | | | | | | | | | | | | | | |
| --- | --- | --- | --- | --- | --- | --- | --- | --- | --- | --- | --- | --- | --- | --- |
| In the current pathway the hearing-impaired patient first visits the ENT-specialist or the AC. Here he is examined and receives a prescription for a hearing aid. Next, the hearing aid dispenser and the patients start a hearing aid trial, where the patient can try several hearing aids. When the patient and dispenser are satisfied with the hearing aid, the patient visits the ENT-specialist or AC to evaluate the hearing aid. | | | | | | | | | | | | | | |
|  | | | | | | | | | | | | | | |
| 1. If you were asked to value this pathway with a grade from 1 to 10, with 10 being the most favourable, what grade would you give? | | | | | | | | | | | | | | |
|  |  |  | | | | | | | | | | | | |
|  | | | |  | |  | |  | |  | |  | |  |
| In the new pathway the hearing-impaired patient first visits the hearing aid dispenser. The dispenser examines whether the patient needs medical care. When necessary the dispenser refers the patient to an ENT-specialist or AC. When the patients is not in need of medical care, the dispenser and patient directly start the hearing aid trial, without prescription. When the patient and dispenser are satisfied with the hearing aid, the patient can go home without having his hearing aid evaluated by an ENT-specialist or AC. | | | | | | | | | | | | | | |
|  | | | | | | | | | | | | | | |
| 1. If you were asked to value this pathway with a grade from 1 to 10, with 10 being the most favourable, what grade would you give? | | | | | | | | | | | | | | |
|  |  |  | | | | | |  | |  | |  | |  |
|  | | | |  | |  | |  | |  | |  | |  |
| Could you please indicate whether you agree with the following statements? | | | | | | | | | | | | | | |
|  | | | Totally agree | | Quite  agree | | Neither agree nor disagree | | Quite disagree | | Totally disagree | | Don’t know | |
| 1. I expect that the hearing aid dispenser will fit a good hearing aid without involvement of the ENT-specialist or AC | | | ❑ | | ❑ | | ❑ | | ❑ | | ❑ | | O | |
|  | | |  | |  | |  | |  | |  | |  | |
| 1. I find it important that the hearing aid is advised by an ENT-specialist or AC | | | ❑ | | ❑ | | ❑ | | ❑ | | ❑ | | **O** | |
|  | | |  | |  | |  | |  | |  | |  | |
| 1. I find it important that the hearing aid is evaluated by an ENT-specialist or AC | | | ❑ | | ❑ | | ❑ | | ❑ | | ❑ | | **O** | |

Final questionnaire for persons with hearing complaints
